# Divergent Effects of a Transient Corticosteroid Therapy on Virus-Specific Quiescent and Effector CD8^+^ T Cells

**DOI:** 10.3389/fimmu.2019.01521

**Published:** 2019-07-12

**Authors:** Dhaneshwar Kumar, Sharvan Sehrawat

**Affiliations:** Department of Biological Sciences, Indian Institute of Science Education and Research Mohali, Mohali, India

**Keywords:** CD8^+^ T cells, virus immunology, immunosuppresants, memory, differentiation

## Abstract

We investigated the influence of a transient treatment of corticosteroid on CD8^+^ T cells during herpesvirus infection. Dexamethasone, a synthetic corticosteroid, induced apoptosis of naïve and memory CD8^+^ T cells but virus-specific effector cells were spared. CD8^+^ T cell susceptibility was directly correlated with the expression of nr3c1. Both α-(HSV1) and γ-(MHV68) herpesvirus infection expanded CD8^+^ T cells down regulated nr3c1 indicating corticosteroid-mediated effects were not limited to one pathogen or the specific clonotype. Dexamethasone compromised anti-viral immunity to subsequent infections, likely through reductions in the naïve cell pool. Dexamethasone augmented the function and inflammatory tissue homing potential of effector cells via upregulation of CXCR3. Accordingly, an antibody neutralization of CXCR3 diminished dexamethasone-induced migration of CD8^+^ T cells to tissues resulting in increased virus burden. Our study therefore suggests that even a transient corticosteroid therapy influences both ongoing CD8^+^ T cell responses as well as the size of the naïve and memory repertoire.

## Introduction

Glucocorticoids cause immunosuppression and are commonly used to ameliorate inflammation resulting from infections, autoimmune diseases, leukemia as well as during transplantation procedures to avoid graft rejection (1–3). Synthetic analogs of glucocorticoids such as dexamethasone, prednisolones are clinically used due to their resistance to host enzymes such as 11 β-hydroxysteroid dehydrogenase-2 (11β-HSD2) that can efficiently inactivate endogenous glucocorticoids. Both endogenous and synthetic glucocorticoids act through intracellular glucocorticoid receptor (GR) encoded by *nr3c1* gene. GRs normally reside in cytosol but translocate to nucleus upon their binding to ligands to effect transcriptional regulation (2, 4, 5). Glucocorticoids have pleiotropic effects on most cell types and organ systems (4). Glucocorticoids induced during infections, cancer progression and various stress responses are involved in regulating neuroendocrine processes via hypothalamic pituitary adrenal axis (HPA) and maintain homeostasis (6). During some systemic herpesviruses and influenza virus infection, the HPA axis modulates disease severity by balancing the immunity and immunopathological responses (7–9). Some of the known immunosuppressive effects of corticosteroids include a modulation of cytokine production by immune cells, an altered cellular trafficking, enhanced phagocytosis as well as the promotion of regulatory T cell function (2, 10). How corticosteroids dictate the fate and function of virus-specific CD8^+^ T cells still remain less well-explored.

As synthetic analogs of glucocorticoids are commonly used to reduce inflammatory responses during herpesvirus infections, we focused to measure the influence of such a therapy on CD8^+^ T cells using dexamethasone as a candidate drug. CD8^+^ T cells are critically involved in controlling the primary infection by herpesviruses as well as maintaining viral latency (11–14).

We demonstrate a tight regulation of nr3c1 during the differentiation of CD8^+^ T cells during herpesvirus infection. Both α- (HSV1) and γ- (MHV68) herpesvirus infection expanded CD8^+^ T cells down regulated their nr3c1 expression in the acute phase of response but memory cells regained the expression. Nr3c1 expression levels in CD8^+^ T cells enhanced the susceptibility of both naïve and memory cells to dexamethasone-induced apoptosis leading to a skewed virus-specific effector CD8^+^ T cell response. Dexamethasone mediated preferential killing of naïve CD8^+^ T cells compromised host's ability to generate an anti-viral CD8^+^ T cell response to a subsequent infection. The residual CD8^+^ T cells however became more functional and preferentially homed to inflammatory tissues due to the transcriptionally controlled upregulation of CXCR3 and CD103. Our study therefore suggests for a limited utility of immunosuppressive corticosteroids in managing inflammatory diseases. Furthermore, corticosteroids could in fact make the host more prone to subsequent infections by limiting naïve and memory CD8^+^ T cells repertoire.

## Materials and Methods

### Mice and Viruses

C57BL/6, TCR Tg OT1xRag1^−/−^ and CD45.1 mice were procured from Jackson laboratory, USA and bred in individual ventilated caging system at the Small Animal Facility for Experimentation (SAFE), IISER Mohali. HSV1-KOS and MHV68-M2-SIINFEKL viruses were grown and titrated using Vero cells (ATCC) as described earlier (15). Institutional Animal Ethics Committee (IAEC) of IISER Mohali set up by the Committee for the Purpose of Control and Supervision of Experiments on Animals (CPCSEA), which is established under Chapter 4, Section 15(1) of the Prevention of Cruelty to Animals Act 1960. IAEC approved all the animal experimental protocols. The protocol numbers were IISERM/SAFE/PRT/2016/09 and 011. All the procedures were performed strictly according to the approved protocols.

### Antibodies, T Cells Isolation Kits and Other Pharmacological Reagents

MHC class I tetramers used in this study were synthesized in house and the monomers were refolded with SIINFEKL and SSIEFARL peptide as described earlier (15–17). Dexamethasone, mifepristone, 2-2-2- tribromoethanol, hematoxylin, and eosin Y were purchased from Sigma Aldrich. Tissue OCT compound was procured from Fischer Scientific. CD8^+^ T cell untouched Dynabeads kits, carboxyfluorescein succinimidyl ester (CFSE), Intracellular fixation-permeabilization buffer, annexin V, and streptavidin-PE were purchased from thermofisher. Antibodies against CD45.1 (A20), CD45.2 (104), CD8 (53-6.7), CD44 (IM7), KLRG1 (2F1), CD11b (M1/70), and TNF-α (TN3-19) and the propidium iodide were purchased from BD pharmingen. Antibodies against CD45 (30-F11), CD69 (H1.2F3), CD11c (N418), PD1 (J43.1), CD16/32 (2.4G2), CD127 (A7R34), and IFN-γ (XMG1.2) were purchased from Tonbo Biosciences. Monensin, Brefeldin A, purified anti-CD3 (17A2), and anti-CD28 (37.51), antibodies against CXCR3 (173), CXCR4 (2B11), CCR7 (4B12) and CD103 (2E7) were procured from eBioscience. CXCR3 neutralizing antibody (CXCR3-173) was purchased from BioXcell.

### Infection of Mice and Dexamethasone Administration

C57BL/6 mice were infected with 5 × 10^5^ pfu of HSV1-KOS by injecting 25 μl of the inoculum per footpad. WT C57BL/6 are less susceptible to HSV1 induced neurological diseases. Moreover, the indicated dose of HSV1-KOS is unlikely to cause neuropathological conditions in more susceptible WT BALB/c mice (18). Mice in one group were injected with the diluent while the other groups were administered with different doses of dexamethasone (10, 0.1, 0.01 mg/Kg B Wt) on day 4, 5, and 6. The response of different cell types was measured 7 dpi. In additional experiments C57BL/6 mice were transferred with indicated number of OT1 cells followed by their infection with MHV68-M2-SIINFEKL via intraperitoneal (5 × 10^5^ pfu) or intranasal (1 × 10^5^ pfu) routes. The cellular responses were analyzed in the spleens and mediastinal LNs for animals infected through intraperitoneal and intranasal routes, respectively.

### Cell Staining for Flow Cytometry

Blood samples were collected from different animals in heparin containing micro centrifuge tubes. Fifty microliter blood was aliquoted directly in a centrifuge tube and 10 μl of antibody mix along with SSIEFARL-tetramers were added. The cells were then incubated at 4**°**C for 45 min in dark. After incubation, 640 μl of 1x ACK solution (155 mM NH_4_Cl, 12 mM NaHCO_3_, 0.1 mM EDTA, pH-7.3) was added to lyse RBCs. The cocktail was kept at room temperature for 15 min. followed by addition of 600 μl PBS. The cells were centrifuged at 1,300 rpm at 4**°**C for 5 min. After two more washings in cold PBS, the cells were resuspended in 200 μl of PBS. A 100 μl of total volume was acquired and analyzed by flowcytometry. Similarly, HSV1 infected mice were treated with dexamethasone or diluent. The peripheral blood samples and PLNs were collected at 6 and 7dpi followed by their cellular phenotypic characterization by flow cytometry using a BD C6 flow cytometer.

### Measuring the Influence of Neutralizing Anti-CXCR3 Antibody on Cellular Migration to Inflammatory Tissues in Dexamethasone Treated Mice

10 × 10^3^ OT1 cells (from OT1 TCR tg mice) were adoptively transferred in C57BL/6 mice, followed by their intranasal infection the next day with MHV68-M2-SIINFEKL (1 × 10^5^ pfu). The animals were then divided into three groups. One group was injected with dexamethasone and an anti-CXCR3 neutralizing antibody on 6 and 7 dpi and other group only received dexamethasone. The lymphoid organs, bronchoalveolar lavage (BAL) and lung tissues were analyzed for cellular distribution and viral load determination at 8 dpi.

### Intracellular Cytokine Staining (ICCS) of CD8^+^ T Cell

Single cell suspensions were prepared from the lymphoid organs of HSV1 or MHV68-M2 SIINFEKL infected animals. 1 × 10^6^ cells were incubated with or without their 1 μg/ml of cognate peptides (SSIEFARL or SIINFEKL), 20 ng/ml of IL-2 and 1x brefeldin A for 4 h. After incubation period was over, the cells were surface stained with anti-CD8 and anti-CD44 antibodies as described earlier and fixed in intracellular fixation buffer for 25 min at room temperature and were then added with 1x permeabilization buffer for 5 min at RT. After 2 more washings an anti-CD16/32 antibody was added for Fc blocking. The cells were then stained with anti-IFN-γ and anti-TNF-α antibodies for 25 min in dark at RT. To remove unbound antibodies, cells were washed thrice and acquired using a flow cytometer.

### Degranulation Staining

For measuring the degranulation of CD8^+^ T cells, an anti-CD107a antibody (1:500 dilution) was added to the SSIEFARL peptide pulsed cells with 1x monensin solution in the complete RPMI. Similarly, control cells were cultured without SSIEFARL peptide. After 4 h of incubation, the cells were surface stained with anti-CD8 and HSV1 H-2K^b^-gB_498−505_ tetramers at 4**°**C in dark for 45 min. Thereafter, cells were acquired by a flow cytometer for degranulation.

### Analysis of mRNA Expression for Different Molecules in CD8^+^ T Cell Subsets Upon Dexamethasone Treatment

1 × 10^5^ OT-1 cells were adoptively transferred into sex-matched C57BL/6 mice. The recipient mice were intraperitoneally infected the following day using 5 × 10^5^ pfu of MHV68-SIINFEKL virus. At 9 dpi, spleens were collected from euthanized mice to prepare single cell suspension. The cells were then stained with fluorescent H-2K^b^-SIINFEKL-tetramers, anti-CD44 and anti-CD8 antibodies. The stained cells were FACS sorted for preparing RNA to measure the expression of different transcripts. Similarly, endogenous H-2K^b^-SSIFARL-tetramer^+ve^ cells recruited in response to HSV1 infection were analyzed for mRNA analysis for different genes. RNA was isolated from sorted CD8^+^ T cells using a kit from Promega (Cat. No. Z6011). The mRNA thus obtained was treated with DNAse followed by its cDNA synthesis using a 50 ng of RNA and SuperScript IV first strand synthesis system kit from Invitrogen (Cat. No.18091050). Qualitative real time PCR (qPCR) reaction was performed using 2x-DyNamo ColorFlash SYBR Green qPCR kit from Thermofisher (Cat. F416L). The reaction was carried out using QuantStudio Real-Time PCR system from Thermofisher. The expression of GAPDH gene served as endogenous control and the relative expression of different genes was calculated by 2^−ΔΔ*CT*^ values. The reaction conditions used for qPCR were: initial denaturation (95**°**C for 7 min), denaturation (95**°**C for 10 s), annealing and extension (60**°**C for 30 s) for 40 cycles. Subsequently melt curve analysis was performed. The primers used and the products size for different genes were; nr3c1 (FP: 5′-AGTGATTGCCGCAGTGAAAT-3′ & RP: 5′-GCCATGAGAAACATCCATGA-3′) = 105 bp, Tbet (FP: 5′ CAATGTGACCCAGATGATCG 3′ & RP: 5′ GCGTTCTGGTAGGCAGTCAC 3′) = 168 bp, Eomesodermin (FP: 5′ TCCTAACACTGGCTCCCACT 3′ & RP 5′ GTCACTTCCACGATGTGCAG 3′) = 153 bp, PD1 (FP: 5′ CCAAGGAACCTGCTTTTCAA 3′ & RP: 5′ GGCATTaCTTGGGAACTGTGT 3′) = 144 bp, Bcl2 (FP: 5′ GCAGATTGCCCTGGATGTAT 3′ & RP: 5′ AGAAAAGTCAGCCAGCCAGA 3′) = 156 bp, CXCR3 (FP: 5′ TACCTTGAGGTTAGTGAACGTCA 3′ & RP: 5′ CGCTCTCGTTTTCCCCATAATC 3′) = 100 bp, GAPDH (FP: 5′-AAATGGTGAAGGTCGGTGTGAAC-3′ & RP: 5′-CAACAATCTCCACTTTGCCACTG-3′) = 90 bp.

### *Ex vivo and in vivo* Assays to Measure Differential Killing of CD8^+^ T Cell Subsets

C57BL/6 female mice were infected with 5 × 10^5^ pfu of HSV1-KOS per footpad. Draining popliteal lymph nodes (PLNs) were collected at 6 or 30 dpi to prepare single cell suspension. Single cell suspensions thus prepared were incubated with graded doses of dexamethasone (0.1–10 μM) in the presence or absence of 5 μM mifepristone, a specific inhibitor for dexamethasone. Subsequently, the kinetics of cell killing activity by 1 μM of dexamethasone in control cells or those pretreated with 5 μM of mifepristone was measured. Diluent controls were invariably included in such experiments. The mifepristone treatment was preceded 1 h by dexamethasone treatment. Cell death was measured by staining cells with annexin V at indicated time points. For *in vivo* apoptosis assays, naive CD8^+^ T cells were isolated from CD45.1 mice using untouched Dynabeads and were subsequently activated with 1 μg/ml of anti-CD3 (plate bound) and anti-CD28 (soluble) antibodies at 37**°**C in CO_2_ incubator for 20 h. Thereafter, some cells were measured for their activation profile using anti-CD69 antibody and the remaining cells were stained with a low concentration of CFSE (0.1 μM). Simultaneously, naive CD8^+^ T cells isolated and purified from CD45.1 mice were stained with a high concentration of CFSE (5 μM). Activated (CFSE^lo^) and naive (CFSE^hi^) CD8^+^ T cells were admixed in 1:1 ratio and a total of 4 × 10^6^ cells were intravenously injected in sex-matched CD45.2 mice. After 30 min of cell transfer one group of animals was treated with 10 mg/kg of dexamethasone and another group with diluent. After 36 h of treatment the frequencies of transferred (CD45.1^+ve^) naive and activated CD8^+^ T cells were measured in different lymphoid organs and peripheral circulation from sham and dexamethasone treated animals. Equivalent numbers of naive (CFSE^hi^) CD8^+^ T cells and activated (CFSE^lo^) CD8^+^ T cells were injected and determined post transfer. The altered frequencies of naive cells and activated CD8^+^ donor T cells in control and dexamethasone treated mice was determined as of their relative frequencies and represented as ratio in different lymphoid organs. Ratio (activated/naïve) = % activated CD8^+^ T cells/% naïve CD8^+^ T cells.

### Measuring the Influence of Dexamethasone on Immune Cell Population in HSV 1 Infected Animals

C57BL/6 female mice were infected in footpad with 5 × 10^5^ pfu of HSV1-KOS as described earlier. Six mice were treated intraperitoneally with dexamethasone (10 mg/Kg B Wt), whereas six mice received diluent on 4, 5, and 6 dpi. To determine the cellular distribution and phenotype in peripheral blood circulation, blood samples were collected. The PBMCs were stained for different surface molecules using fluorochrome labeled antibodies and were then analyzed by Accuri C6 flow cytometer. At 7 dpi all the animals were sacrificed and draining PLNs were processed to isolate cells which were then analyzed by H-2K^b^-SSIEFARL-tetramer for antigen-specific CD8^+^ T cells. The functionality of the cells was determined by intracellular cytokine staining assays (ICCS) for the production of cytokines. Surface staining for different molecules was performed by flow cytometry. The virus titers in the footpad tissues were measured by plaque forming assays.

For some experiments, we *in vitro* stimulated OT1 cells with anti-CD3 and anti-CD28 for 16 h and thereafter a fraction of these cells were treated with 1μM of dexamethasone and the other fraction with only the diluent for 1 h. The cells were then washed extensively and transferred in separate groups of animals to measure the survival of these cells. The recipient animals were then analyzed for analyzing donor cells for different parameters. A recall response of the surviving cells was analyzed by infecting recipient animals via intranasal route with MHV68-M2-SIINFEKL after 30 days of transfer. The expanding cells were analyzed 8 days later by flow cytometry. The lung samples were analyzed histologically and the virus titration.

### Measuring the Responsiveness of Dexamethasone Pre-treated Animals to a Subsequent Infection

Sex-matched CD45.1 C57BL/6 mice received 10,000 naive and purified OT-1 cells. The next day these mice were injected with CFA in hind footpad to induce the non-specific inflammation. After a dexamethasone (10 mg/Kg body weight) treatment from 4 to 6 dpi and 30 days of cell transfer these mice were infected with 1 × 10^5^ pfu of MHV68-SIINFEKLthrough intranasal route and simultaneously CFA was injected in the hind footpad. At 10 dpi of MHV68 infection the cellular analysis was performed in PBMCs and these mice were sacrificed at 11 dpi to collect BAL, mediastinal lymph nodes and spleens for analysis.

### Plaque Forming Assays

To determine the viral load in mice organs (foot pad and lungs), Vero cells were plated in 24 well plates. Next day, 90–100% confluent cells were added with the supernatant of tissue homogenates. The supernatants were removed 2 h later and low melting agar prepared in 10% DMEM (2x) was gently poured on the surface of cells. The plates were incubated at 37**°**C for 4–5 days in CO_2_ incubator. Once plaques were visible under microscope cells were fixed with 10% paraformaldehyde overnight at RT. Next day the fixation buffer and agar plugs were removed to stain the fixed cells with 0.2% crystal violet and the numbers of plaques were counted for each dilution. The number of plaque forming units per ml of solution were calculated as:

pfu/ml = (No. of plaques/volume plated) × dilution factor

pfu/ml in target tissue was represented as pfu/gm of the tissue.

### Statistical Analysis

Graph pad prism software version 7 was used for statistical analysis. Data with similar variances and having Gaussian distribution were analyzed with two tailed unpaired Student's *t*-tests. Data not following Gaussian distribution were analyzed with two tailed Mann-Whitney U tests. For multiple comparisons two-way ANOVA test with Bonferroni *post hoc* analysis were used. The *p*-value below 0.05 considered as significant. ^*^ < 0.05, ^**^ < 0.01 and ^***^ < 0.001.

## Results

### Dexamethasone Alters the Distribution of CD8^+^ T Cells During Herpesvirus Infection

Dexamethasone is a commonly used immunosuppressant for controlling inflammation caused by infections. We investigated the influence of such a transient therapy on differentiation program of virus-specific CD8^+^ T cells in HSV1 infected mice. While the frequencies and numbers of leukocytes (CD45^+^ cells) were reduced by 2 fold in the peripheral circulation of infected mice receiving corticosteroid therapy as compared to controls, the frequencies of CD11b^+^ myeloid cells increased by more than 2 fold (Figures S1A–E).

**Figure 1:**
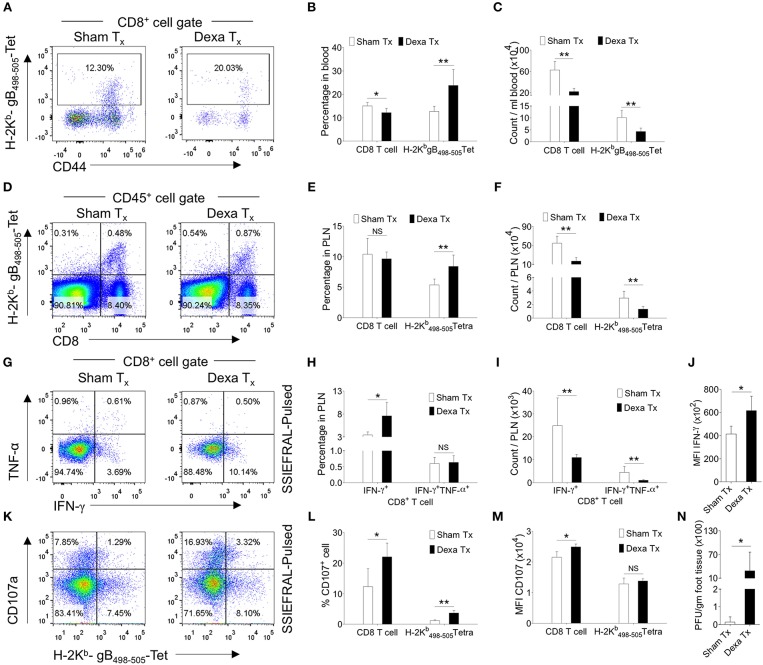
Dexamethasone therapy induces altered response in HSV1 infected animals. C57BL/6 mice were infected with HSV1-KOS (5 × 10^5^ pfu/foot pad) and were then injected with the diluent or dexamethasone (10 mg/Kg Bwt) intraperitoneally from 4 to 6 dpi. Distribution and phenotype of immune cells were analyzed in the peripheral blood circulation at 6 dpi **(A–C)** and draining popliteal lymph node (PLN) at 7 dpi **(D–M)**. **(A)** Representative FACS plots from two groups show the frequency and phenotype of HSV-1 specific (H-2K^b^-gB_498−505_-tetramer^+ve^) CD8^+^ T cells in the peripheral circulation. **(B)** Bar diagrams show the frequencies and absolute numbers of CD8^+^ T cells out of CD45^+^ leukocytes and HSV1 specific (H-2K^b^-gB_498−505_-tetramer^+ve^) CD8^+^ T cells in peripheral blood. **(C)** Bar diagrams show the numbers of CD8^+^ T cells and HSV1-specific (H-2K^b^-gB_498−505_-tetramer^+ve^) CD8^+^ T cells per ml of blood. **(D)** Representative FACS plots from two groups show the frequencies of HSV1 specific (H-2K^b^-gB_498−505_-tetramer^+ve^) CD8^+^ T cells among CD45^+^ leukocytes present in draining popliteal LNs. **(E)** Percentage of total CD8^+^ T cells and HSV1 specific (H-2K^b^-gB_498−505_-tetramer^+ve^) CD8^+^ T cells among CD45^+^ leukocytes present in draining popliteal LNs are shown by bar diagrams. **(F)** Total numbers of CD8+ T cells and HSV1 specific (H-2K^b^-gB_498−505_-tetramer^+ve^) CD8^+^ T cells among CD45^+^ leukocytes present in draining popliteal LNs are shown by bar diagrams. **(G–J)** ICCS assays were performed by stimulating PLN cells with SSIEFARL peptide to measure cytokine producing CD8^+^ T cells in the PLNs of HSV1 infected and dexamethasone treated animals. The percentages and numbers of cytokine producing CD8^+^ T cells in PLN are shown by representative FACS plots **(G)** and bar diagrams **(H)**. **(I)** Total numbers of CD8^+^ T cells that produced indicated cytokines are shown by bar diagrams. **(J)** The mean fluorescence intensities (MFI) for IFN-γ produced by peptide stimulated CD8^+^ T cells from control and dexamethasone group are shown by bar diagrams. Representative FACS plots **(K)** and bar diagrams **(L,M)** show the frequencies and numbers of de-granulating (CD107a^+^) CD8^+^ T cells isolated from dexamethasone and control groups of animals. **(M)** The mean fluorescence intensities (MFI) for CD107a by CD8^+^ T cells from control and dexamethasone group are shown by bar diagrams. **(N)** Viral loads measured from the extracted footpads of sham and dexamethasone treated mice are shown by bar diagram. Mean values and ± SD are shown. In each experimental group 4–5 animals were included. The experiments were repeated with similar results. **p < 0.005; *p < 0.05 and NS (*p* > 0.05)- not significant (Mann-Whitney U test- two tailed).

Similarly, in comparison to controls, dexamethasone treated mice had 2 fold higher frequencies of H-2K^b^-gB_498−505_-tetramer^+ve^ CD8^+^ T cells but their total numbers were lower (Figures 1A–C). Similar results were recorded for cell distribution in the draining PLN of treated group as compared to controls (Figures 1D–F). We also measured the frequencies and total number of cytokine producing CD8^+^ T cells responding to the immunodominant H-2K^b^ restricted gB_498−505_-(SSIEFARL) peptide of HSV1, using intracellular cytokine staining (ICCS) assays and essentially similar results were obtained for IFN-γ producing CD8^+^ T cells as well as its per cell basis expression (Figures 1G–J). However, the frequencies of TNF-α^+^CD8^+^ T cells remained unaltered in two groups (Figures 1G–J). We also measured the frequencies of degranulating (CD107a^+^) CD8^+^ T cells in response to SSIEFARL peptide and such were more abundant in dexamethasone treated animals as compared to controls (Figures 1K–M). The enhanced expression of CD107a and the cytokine producing ability of CD8^+^ T cells are associated with their superior functionality (19). Our data therefore suggested that dexamethasone enhanced the function of herpesvirus specific CD8^+^ T cells. We also assessed whether or not a better functionality of surviving virus-specific CD8^+^ T cells contributes to an efficient viral control by quantifying virus loads in the footpad tissues collected from the two groups. Although more replicating viral particles were found in dexamethasone treated animals at 7 dpi; by 10 dpi all the animals cleared the replicating virus (Figure 1N, data not shown). Experiments aimed at measuring the dose response effect of dexamethasone on anti-viral CD8^+^ T cells revealed that upto a 100 fold lower dose (0.1 mg/Kg B Wt) did not significantly change either the function or the phenotype of different CD8^+^ T cell subsets and hence the observed effects of dexamethasone were highly dose dependent (Figure S2, data not shown). Therefore, we concluded that the dexamethasone enhanced the frequencies but not the absolute numbers of virus-specific CD8^+^ T cells that resulted in a delayed viral control.

### Dexamethasone Promotes CXCR3 Mediated Migration of CD8^+^ T Cells to Inflammatory Sites

**Figure 2:**
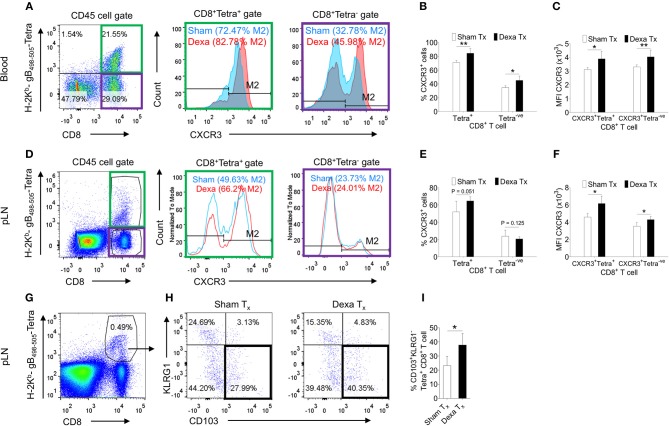
Dexamethasone modulates tissue homing molecules in CD8^+^ T cells during HSV1 infection. C57BL/6 mice were infected with HSV1-KOS (5 × 10^5^ pfu/foot pad) and were then injected with the diluent or dexamethasone (10 mg/Kg Bwt) intraperitoneally from 4 to 6 dpi. Expression levels of CXCR3 on CD8^+^ T cell subsets in blood (6 dpi) and draining popliteal LNs (7 dpi) isolated from diluent and dexamethasone treated mice were measured by flow cytometry. Representative FACS plots shows CXCR3 expression on H-2K^b^-gB_498−505_-tetramer^+ve^ and H-2K^b^-gB_498−505_-tetramer^−ve^CD8^+^ T cells in blood circulation **(A)** and draining PLN **(D)**. **(B)** The percentage of CXCR3^+^ H-2K^b^- gB_498−505_-tetramer^+ve^ and CXCR3^+^ H-2K^b^- gB_498−505_-tetramer^−ve^ cells is shown by bar diagrams. **(C)** Mean fluorescence intensities (MFI) for CXCR3 expression by H-2K^b^- gB_498−505_-tetramer^+ve^ and H-2K^b^-gB_498−505_-tetramer^−ve^CD8^+^ T cells are shown by bar diagrams. **(E)** The percentage of CXCR3^+^ H-2K^b^-gB_498−505_-tetramer^+ve^ and CXCR3^+^ H-2K^b^- gB_498−505_-tetramer^−ve^ cells is shown by bar diagrams. **(F)** MFI for CXCR3 on H-2K^b^-gB_498−505_-tetramer^+ve^ and H-2K^b^-gB_498−505_-tetra^−ve^CD8^+^ T cells are shown by bar diagrams. **(G–I)** The frequencies CD103^+^ KLRG1^−^ T cells out of H-2K^b^-gB_498−505_-tetramer^+ve^CD8^+^ T cells in sham and dexamethasone treated mice infected with HSV1 are shown by representative FACS plots **(G,H)** and bar graphs **(I)** in the draining popliteal LNs cells. Mean ± SD are represented. The experiments were repeated two times with similar results. In each experimental group 4–6 animals were included. **p < 0.005; *p < 0.05, and NS (*p* > 0.05)- not significant (Unpaired *t*-test).

Dexamethasone enhanced the frequencies but not numbers of virus-specific CD8^+^ T cells; we therefore tested whether such an effect could either be due to their distinct homing pattern or their differential survival or both. Some reports in fact suggested for a modulation of CXCR4 expression pattern by naive T cells and monocytes responding to corticosteroids (20, 21). We measured the expression of a number of homing molecules such as the CXCR3, CXCR4, CCR7 and CD103. CXCR3, CXCR4, CD103 induce a preferential migration of CD8^+^ T cells to tissue sites while CCR7 expression help retain such cells in lymphoid organs (22, 23). The MFI values and the frequencies of CXCR4 (~11% in PBMCs and ~6% in PLN) and CCR7 (~1% in PBMCs and 45% in PLN) expressing H-2K^b^-gB_495−505_-tetramer^+ve^CD8^+^ T cells were similar in control and treated mice (Figure S3). The frequencies of CXCR3^+^H-2K^b^-gB_495−505_-tetramer^+ve^CD8^+^ T cells were significantly higher in the peripheral blood and PLNs of dexamethasone treated mice (83% in PBMCs and 66% in PLN) as compared to controls (72.4% in PBMCs and 49% in PLN) and similar results were recorded for its MFI values (~1.5 to 2 fold higher) (Figures 2A–F). Interestingly, the proportions of CXCR3^+^K^b^-gB_495−505_-tetramer^−ve^CD8^+^ T cells were also greater in dexamethasone (46%) as compared to control (33%) group (Figures 2A,B). Only a moderate change in the percentage of CXCR3^+^CD8^+^ T cells in draining PLN could indicate that a majority of CXCR3^+^CD8^+^ T cells might have migrated from LNs to tissue sites. A further phenotypic analysis of virus-specific CD8^+^ T cells in HSV1 infected mice revealed a greater proportions of KLRG1^−ve^CD103^+ve^K^b^-gB_498−505_-tetramer^+ve^CD8^+^ T cells in the draining LNs of dexamethasone treated animals as compared to those in controls (~1.5 fold higher, Figures 2G,H). Some earlier studies showed that activated CD8^+^ T cells with the observed phenotype preferentially generate tissue resident memory precursor cells (24).

**Figure 3:**
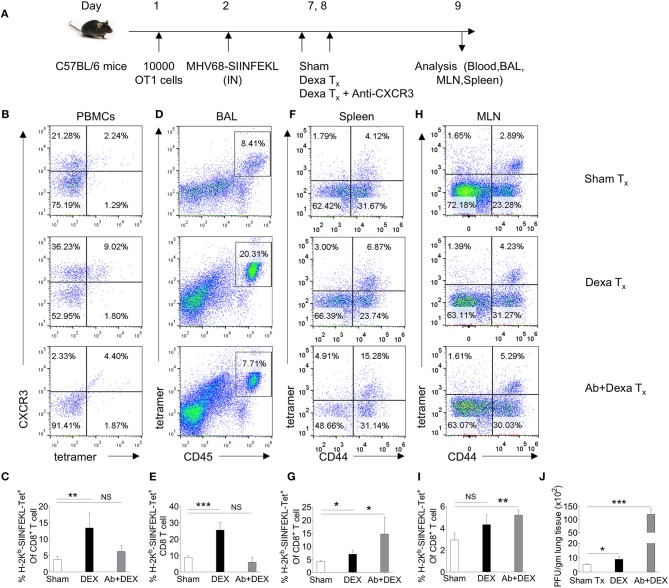
A transient dexamethasone therapy induces CXCR3 mediated CD8^+^ T cells migration to tissue sites. **(A)** A schematic of the experiments is shown. 10 × 10^3^ OT1 cells were transferred in C57BL/6 mice. Recipient animals were infected intranasally with MHV68 M2-SIINFEKL virus the following day. Infected animals were then divided into three groups. One group received diluent. The second group received dexamethasone (10 mg/kg B Wt) and the third group received both dexamethasone and anti-CXCR3 antibody at day 6 and 7 post infection. The response of CD8^+^ T cells and viral load were analyzed at 8 dpi. **(B,C)** The frequencies and phenotype of H-2K^b^-SIINFEKL-tetramer^+ve^CD8^+^ T cells are shown by representative FACS plots **(B)** and bar diagrams **(C)** in the peripheral blood of animals in different groups. **(D,E)** The frequencies of H-2K^b^-SIINFEKL-tetramer^+ve^CD8^+^ T cells are shown by representative FACS plots **(D)** and bar diagrams **(E)** in bronchoalveolar tissue lavage samples of animals from different groups. **(F,G)** The frequencies and phenotype of K^b^-SIINFEKL-tetra^+ve^CD8^+^ T cells are shown by representative FACS plots **(F)** and bar diagrams **(G)** in the draining mediastinal LNs of animals in different groups. **(H,I)** The frequencies and phenotype of H-2K^b^-SIINFEKL-tetramer^+ve^CD8^+^ T cells are shown by representative FACS plots **(H)** and bar diagrams **(I)** in the spleens of animals in different groups. **(J)** Viral load was measured from lung homogenates of different groups of animals by plaque forming assays. The replicating virus load is shown by bar diagrams in different groups. In each experimental group 5 animals were included. ***p < 0.001; **p < 0.005; *p < 0.05, and NS (*p* > 0.05)- not significant (Unpaired *t*-test).

We performed further experiments to elucidate whether dexamethasone induced up regulation of CXCR3 on CD8^+^ T cells facilitates their migration to inflammatory tissues by administering a neutralizing anti-CXCR3 antibody (25) (Figure 3A). A homogenous population of TCR transgenic T cells, OT1 cells were transferred to animals prior to their infection with a mutant γ-HV (MHV68-M2-SIINFEKL), encoding the cognate peptide presented by H-2K^b^ to such cells. As compared to control animals, dexamethasone treated mice had higher frequencies (upto 3 fold) of H-2K^b^-SIINFEKL-tetramer^+ve^CD8^+^ T cells both in the peripheral blood (4 vs. 13%) and BAL tissues (9 vs. 26%) (Figures 3B–I). An administration of anti-CXCR3 antibody along with dexamethasone drastically reduced their frequencies in both the compartments i.e., peripheral blood and BAL (Figures 3B–E). The antibody therapy simultaneously increased the frequencies of H-2K^b^-SIINFEKL-tetramer^+ve^CD8^+^ T cells in spleens and draining mediastinal LNs (Figures 3F–I). The replicating virus in the lung tissues increased more than 20 fold in antibody-injected animals (Figure 3J).

Taken together our results demonstrated that dexamethasone enhanced the expression of chemokine receptors, CXCR3, on CD8^+^ T cells that facilitated their migration to the infection sites to effect a better replicating virus control.

### Dexamethasone Preferentially Induces Apoptosis of Naïve CD8^+^ T Cells

**Figure 4:**
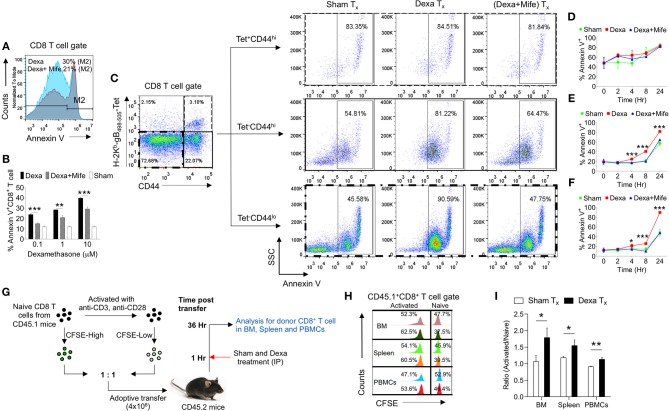
Naive CD8^+^ T cell are more susceptible to dexamethasone-induced cell death. Single cells suspension prepared from the draining popliteal LN cells of HSV1 infected mice were incubated with diluent and dexamethasone for the indicated time points. Some cells were pretreated with 5 μM mifepristone. The frequencies of apoptotic cells were measured by annexin V staining after incubation period was over. **(A)** Representative overlaid histograms show annexin V^+ve^ cells from dexamethasone and dexamethasone combined with mifepristone treated samples. **(B)** Bar diagrams show the percentage of annexin V^+ve^ cells in different groups. **(C)** Annexin V^+ve^ cells in boxed CD8^+^ T cells (naïve, CD44^+^ H-2K^b^-tetramer^−ve^, and CD44^+^- H-2K^b^-tetramer^+ve^) that were incubated with diluent, dexamethasone (1 μM) and dexamethasone (1 μM) + mifepristone (5 μM) are shown. **(D–F)** Kinetics of annexin V^+ve^ cells in different CD8^+^ T cell subsets is shown. Mean and ± SD are shown. ***p ≤ 0.001, **p ≤ 0.005, *p ≤ 0.05 (Bonferroni test-Two-way ANOVA). **(G–I)** *In vivo* killing of different subsets of CD8^+^ T cells was measured. **(G)** Schematic of the experiments to measure *in vivo* killing of identifiable cells is shown. **(H)** Population of naive (CFSE^hi^) and activated (CFSE^lo^) CD45.1^+^CD8^+^ T cells in different tissues (BM, Spleen and PBMCs) of sham (upper panel) and dexamethasone (lower panel) treated CD45.2 mice are shown by histograms. **(I)** Ratio of activated and naive CD8^+^ T cells in different tissue sites of sham and dexamethasone treated mice is shown by bar diagrams. In each experimental group 6 animals were included Mean and ± SD are shown. ***p ≤ 0.001, **p ≤ 0.005, *p ≤ 0.05 (Unpaired *t*-test).

The observed skewed frequencies of CD8^+^ T cell subsets in the dexamethasone treated mice could also be attributed to their differential survivability. To explore this possibility we isolated draining popliteal LN cells from HSV1 infected animals and exposed them to the graded doses of dexamethasone (0.1, 1, and 10 μM). Some of the cells were pretreated with mifepristone, a specific competitive inhibitor of glucocorticoid receptors (26). Dexamethasone induced apoptosis of CD8^+^ T cells in a dose and time dependent manner and the effects were significantly rescued by a prior treatment with mifepristone (Figures 4A,B). We then measured apoptotic cells by staining with annexin V in different subsets of CD8^+^ T cells. While the virus-specific activated CD8^+^ T cells (H2-K^b^-SIINFEKL-tetramer^+ve^CD44^hi^ CD8^+^ T cells) were not affected by dexamethasone (84.8 ± 3.45% in sham vs. 83.3 ± 2.5% annexin V^+ve^ in dexamethasone gp), a significant proportion of tetramer^−ve^ CD44^lo^CD8^+^ T cells (48.5 ± 7.1% in sham vs. 89.9 ± 1.2% annexin V^+ve^ in dexa group) and tetramer^−ve^CD44^hi^CD8^+^ T cells (57.3 ± 4.7% annexin V^+ve^ cells in sham vs. 81.7 ± 1.2% annexin V^+ve^ cells in dexamethasone group) exhibited enhanced apoptosis (Figures 4C–F). A prior treatment of mifepristone greatly rescued susceptible CD8^+^ T cells from drug-induced apoptosis at all the time points investigated (Figures 4B–F). This suggested that the receptor, nr3c1 ligation was crucial for the observed effects.

In order to test whether or not *e*x *vivo* results could be recapitulated *in vivo*, we measured the dexamethasone induced differential killing of naïve and activated CD8^+^ T cells using an adoptive transfer approach. CD8^+^ T cells were activated *in vitro* using anti-CD3 and anti-CD28 antibodies. The treatment induced the activation of more than 80% cells (CD69^+^) (data not shown). The activated cells and the freshly isolated naïve CD8^+^ T cells (both CD45.1^+ve^) were labeled with high and low concentrations of CFSE (CFSE^hi^ and CFSE^lo^) and their equal numbers were transferred into CD45.2 congenic mice. One group of recipient mice was administered with a single dose of dexamethasone and the surviving CD45.1^+ve^ cells frequencies were measured (Figure 4G). Dexamethasone treated mice significantly reduced the proportions of transferred naïve CD8^+^ T cells as compared to their activated counterparts confirming the results from *in vitro* assays (Figures 4H,I). Varying frequencies of surviving naïve cells in different lymphoid organs could be due to the differential bioavailability of dexamethasone or their homing pattern.

Our results therefore demonstrated that dexamethasone-induced preferential elimination of naïve CD8^+^ T cells in addition to their distinct homing pattern skewed response toward virus-specific CD8^+^ T cells in infected mice.

### A Transient Dexamethasone Therapy Compromised Host's Ability to Mount a Specific CD8^+^ T Cell Response to a Subsequent Infection

**Figure 5:**
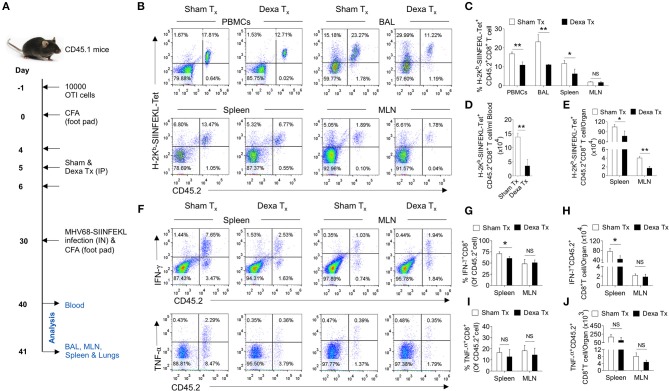
A transient corticosteroid therapy compromises the host's ability to mount an efficient CD8^+^ T cells response to a subsequent infection. **(A)** Schematic of the experiments is shown. Congenic CD45.1 mice were injected 10 × 10^3^ OT1 cells 1 day prior to injecting complete Freund's adjuvant (CFA) to induce a non-specific inflammation in footpads. The animals were treated with dexamethasone or sham from 4 to 6 days of cell transfer. On 30 days, the mice were infected with MHV68-M2 SIINFEKL via intranasal route and simultaneously CFA was injected in the footpad to neutralize the influence of any inflammation induced cellular migration. The response of remaining OT1 cells was measured at 10 and 11 dpi in the peripheral circulation (at 10 dpi) and different lymphoid organs and tissue sites (at 11 dpi). **(B,C)** The frequencies and numbers of SIINFEKL specific CD45.2^+^CD8^+^ T cells in blood circulation, bronchoalveolar lavage (BAL), spleen and mediastinal LNs are shown by representative FACS plots and bar diagrams. **(D)** The numbers of H-2K^b^-SIINFEKL-tetramer^+ve^ cells /ml of blood are shown by bar diagrams. **(E)** The numbers of H-2K^b^-SIINFEKL-tetramer^+ve^ cells in spleen and mediastinal LNs are shown by bar diagrams. **(F–J)** Single cell suspensions prepared from spleens and mediastinal LNs were subjected to ICCS assays to measure SIINFEKL-specific CD8^+^ T cells that produced IFN-γ and TNF-α. The frequencies and numbers of IFN-γ^+^CD8^+^ T cells or TNF-α^+^CD8^+^ T cells in spleen and mediastinal LNs are shown by representative FACS plots **(F)** and bar diagrams **(G–J)**. Bar diagrams shown the frequencies **(G)** and numbers **(H)** of IFN-γ^+^CD8^+^ T cells in spleen and MLN samples. Bar diagrams shown the frequencies **(I)** and numbers **(J)** of TNF-α^+^CD8^+^ T cells in spleen and MLN samples. Mean ± SD for each bar is depicted. In each experimental group 5 animals were included and the experiments were repeated two times. **p < 0.005, *p < 0.05, and ns (*p* > 0.05)- not significant (Mann-Whitney U test- two tailed).

Having established a preferential elimination of naive CD8^+^ T cells by dexamethasone, we tested whether such a situation could compromise the host's ability to generate an efficient antigen-specific CD8^+^ T cells response against a subsequent infection. A schematic of these experiments is shown in Figure 5A. We transferred 10 × 10^3^ OT1 cells (CD45.2^+ve^) in CD45.1^+^ mice 1 day prior to injecting complete Freund's adjuvants (CFA) in their hind footpads. The injection could generate a non-specific inflammatory response as a mimic to clinical situations requiring a corticosteroids therapy. We hypothesized that if indeed a transient administration of dexamethasone reduced the frequencies of naïve OT1 cells, a subsequent MHV68-M2-SIINFEKL infection would expand such cells inefficiently. After a transient dexamethasone or sham treatment from 4 to 6 days post CFA injection the response of residual cells was measured by infecting with MHV68-M2-SIINFEKL. A second injection of CFA in the footpad induced inflammatory response to a distal site from the respiratory tract to regulate any migration of CD8^+^ T cells towards respiratory tract, possibly contributed by their enhanced CXCR3 expression. The frequencies and total numbers of donor H-2K^b^-SIINFEKL-tetramer^+ve^CD8^+^ T cells (CD45.2^+ve^) decreased in the peripheral blood (1.5 fold reduction), BAL (>2 fold reduction) and spleens (a >2 fold reduction) in dexamethasone pre-treated group as compared to the control (Figures 5B–E). Similar results were evident for IFN-γ producing cells in the draining mediastinal LNs (up to 2 fold reduction) and spleens (up to 1.5 fold reduction) of dexamethasone group as compared to the sham treated mice (Figures 5F–H). No significant differences were observed in the frequencies of TNF-α producing CD8^+^ donor T cells in the two groups of animals (Figure 5F, lower panel, Figures 5I,J).

Thus, our results show that dexamethasone induced a preferential elimination of naïve cells whose activity controls a subsequent infection.

### Virus-Specific CD8^+^ T Cells Modulates the Expression of Glucocorticoid Receptor, Nr3c1 During Their Differentiation

**Figure 6:**
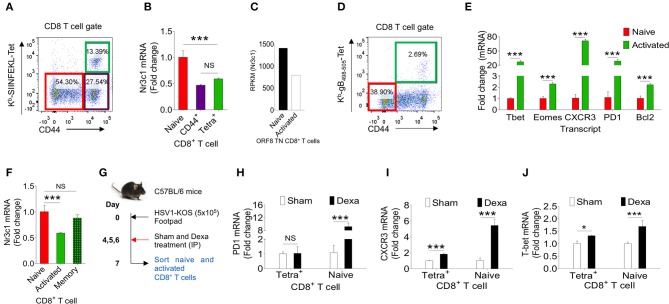
Dexamethasone alters transcriptional profile of virus expanded CD8^+^ T cells. Different subsets of CD8^+^ T cells were FACS sorted from C57BL/6 mice that were transferred with 1 × 10^5^ of OT1 cells and infected intraperitoneally with MHV68-M2-SIINFEKL. Indicated cell populations were FACS sorted. **(A)** Sorted CD8^+^ T cell subsets (naïve-CD44^lo^; H-2K^b^-SIINFEKL tetramer^−ve^CD44^hi^ and H-2K^b^-SIINFEKL-tetra^+ve^CD44^+^) from spleens of MHV68-M2-SIINFEKL infected mice. **(B)** Quantitative RT-PCR data for glucocorticoid receptor (nr3c1) expression in sorted CD8^+^ T cell subsets as shown as fold change of naïve H-2K^b^-SIINFEKL tetramer^+ve^. **(C)** RPKM values of nr3c1 in naïve and activated MHV68 specific TCR transnuclear (TN) CD8^+^ T cells as measured by RNAseq are shown. **(D,E)** HSV1 expanded endogenous CD8^+^ T cells of indicated phenotype were FACS-sorted from the draining popliteal LNs at 7dpi of HSV1 infected mice. **(D)** A representative FACS plot show the sorted cell population. **(E)** RT-PCR was performed for measuring mRNA of indicated gene expression in naive and virus specific (H-2K^b^-gB_498−505_-tetramer^+ve^) CD8^+^ T cell subsets activated and expanded by HSV1 infection. Relative expression as fold change for different gene is shown. **(F)** RT-PCR was performed for measuring the mRNA of nr3c1 gene expression in naïve, HSV1 specific (H-2K^b^- gB_498−505_-tetramer^+ve^) CD8^+^ T cells in the acute (7 dpi) and memory stage (60 dpi) of the response in HSV1 infected mice. The fold change is shown by bar diagram. Experiments were performed with more than 3 animals at each time point. Mean and ± SD are shown. ***p ≤ 0.001, **p ≤ 0.005, *p ≤ 0.05 (Unpaired t test). **(G)** A schematic of the experiments to study the effect of corticosteroid on transcript expression level is shown. **(H–J)** Naïve and activated HSV1 specific (H-2K^b^-SSIEFARL-tetramer^+ve^) CD8^+^ T cells were isolated from control and dexamethasone HSV1 infected animals (7 dpi). The expression levels PD1 **(H)**, CXCR3 **(I)**, and Tbet **(J)** were measured by quantitative RT-PCR. Bar diagrams show the relative fold change in the expression for different genes. Mean ± SD are shown. ***p < 0.001; *p < 0.05 and NS (*p* > 0.05)- not significant (Unpaired *t*-test).

Dexamethasone like other glucocorticoids acts through nr3c1 receptor (4, 5). We, therefore measured its expression in herpesvirus expanded CD8^+^ T cells to account for their differential response to dexamethasone. We analyzed naïve and activated TCR transgenic OT1 cells, TCR transnuclear (TN) MHV68-ORF8 specific cells as well as endogenous CD8^+^ T cells expanded in the course of γ-(MHV68-M2-SIINFEKL) and α-(HSV1) herpesviruses. Different populations were included in the analysis considering the possibilities that varying antigen receptor affinities, the methods for their generation, the existing differences in pathogen specific elements, the microenvironment in which such cells were activated as well as their functional and phenotypic heterogeneity all could contribute to the transcriptional profile of such cells (27). OT1 cells were transferred in C57BL/6 mice followed by infection with MHV68-M2-SIINFEKL. On 10 dpi, subsets of CD8^+^ T cells were FACS sorted. Sorted CD8^+^ T cell population were: CD44^hi^H2-K^b^-SIINFEKL-tetramer^+ve^CD8^+^ T cells, CD44^hi^H-2K^b^-SIINFEKL-tetramer^−ve^CD8^+^ T cells and un-activated CD44^lo^H2-K^b^-SIINFEKL-tetramer^−ve^CD8^+^ T cells (Figure 6A). The purity of different subsets of CD8^+^ T cells were more than 98% (Figure S4A). MHV68 expanded cells down regulated nr3c1 mRNA levels by 1.5 fold as compared to un-activated cells (Figure 6B). MHV68 virus infection expanded transnuclear (TN) CD8^+^ T cells that were specific to an epitope of the wild type virus (H-2K^b^-ORF8-(KNYIFEKL)-tetramer^+ve^CD8^+^ TN T cells) also showed a 2 fold down regulation of nr3c1 expression as compared to their naïve counterparts when measured by RNAseq [Figure 6C, (27)]. The expression of nr3c1 and other molecules associated with CD8^+^ T cell activation and differentiation were also simultaneously measured for endogenous virus-specific CD8^+^ T cells isolated from HSV1 infected mice in the acute phase of their response at 7dpi (Figures 6D,F). The expression levels of Bcl2, an anti-apoptotic molecule, PD1, a molecule that was initially found to be overexpressed by apoptotic cells (28), the chemokine receptor CXCR3 that facilitates cellular migration to inflammatory tissues (29, 30) as well as transcription factors, such as Tbet and Eomes, that control differentiation of CD8^+^ T cells (31) were measured. Tbet also regulates CXCR3 and IFN-γ expression in virus-specific CD8^+^ T cells (30, 32). In the sorted cells the cross-contamination was <0.5% (Figure S4B). The expression of nr3c1 was reduced by ~2 fold in activated H-2K^b^-gB_495−505_-tetramer^+ve^CD44^hi^CD8^+^ T cells as compared to naïve cells but that of Bcl2, PD1, CXCR3, Tbet, and Eomes were enhanced by ~2, 28, 70, 24, and 2 fold in these cells (Figures 6E,F). A fraction of activated cells differentiate to become long-lived memory cells, therefore an up regulation of Bcl2 was not surprizing. Similar results were reported in earlier studies (27, 33–35).

To gain further insights into the differentiation of virus-specific CD8^+^ T cells under the influence of a transient corticosteroid therapy, we measured mRNA levels of different molecules in HSV1 specific (H-2K^b^-gB_495−505_-tetramer^+ve^CD44^hi^) and HSV1 non-responsive (H-2K^b^-gB_495−505_-tetramer^−ve^CD44^lo^) CD8^+^ T cells isolated from infected mice that were either treated with dexamethasone or given a sham treatment (Figure 6G). The cross contamination in sorted cells was <0.5 % (Figure S4B). Although H-2K^b^-gB_495−505_-tetramer^+ve^ CD44^hi^CD8^+^ T cells downregulated nr3c1 expression dexamethosone did not alter its expression further in either cell types (Figure 6F, Figure S4C). The expression levels of Bcl2 and Eomes also did not change significantly in the two cell populations isolated from dexamethasone treated and control animals (Figures S4D,E). Naïve CD8^+^ T cells upregulated PD1 to a staggering 10 fold level but its expression remained unaltered in CD44^hi^H-2K^b^-gB_495−505_-tetramer^+ve^CD8^+^ T cells isolated from control and dexamethasone treated mice (Figure 6H). PD1 expression is induced in TCR or the coreceptor stimulated cells (36). Therefore, an enhanced expression of PD1 in naïve CD8^+^ T cells was rather surprizing and could suggest for their alternate activation program induced by dexamethasone. CXCR3 was also further up regulated both in activated H-2K^b^-gB_495−505_-tetramer^+ve^CD44^hi^CD8^+^ T cells (by 2 fold) and naïve cells (by more than 5 folds) isolated from dexamethasone treated group as compared to sham treated cells (Figure 6I). The transcription factor Tbet, which governs the differentiation of CD8^+^ T cells and also plays a role in controlling CXCR3 was upregulated both in CD44^hi^H-2K^b^-gB_495−505_-tetra^+ve^CD8^+^ T cells (1.3 fold) and naïve cells (more than 2 folds) of dexamethasone treated group as compared control (Figure 6J). These results suggest that dexamethasone induces a specific transcriptional program in naïve and antigen activated cells to exhibit specific phenotype.

Our data therefore suggest that activated CD8^+^ T cells down regulate their nr3c1 expression irrespective of the source, and antigen recognition affinities. Furthermore, dexamethasone could induce an activation phenotype in CD8^+^ T cells irrespective of their antigenic stimulation.

### Preformed Virus Specific Memory CD8^+^ T Cells Undergo Dexamethasone Induced Apoptosis

**Figure 7:**
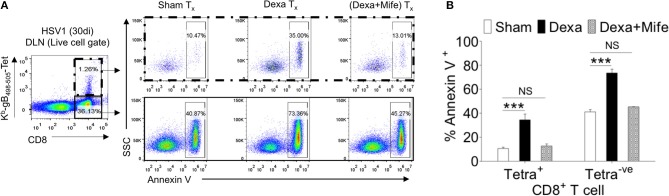
Susceptibility of memory CD8^+^ T cells to dexamethasone. Single cells suspension prepared from the draining popliteal LN cells of HSV1 infected mice (30 dpi) were incubated with diluent and dexamethasone (1 μM) for 8 h. Some cells were pretreated with 5 μM mifepristone. The frequencies of apoptotic cells were measured by annexin V staining after incubation period was over. **(A)** Annexin V^+ve^ cells in boxed CD8^+^ T cells populations (H-2K^b^-SSIEFARL-tetramer^−ve^ and H-2K^b^-SSIEFARL-tetramer^+ve^) that were incubated with diluent, dexamethasone (1 μM), and dexamethasone (1 μM) + mifepristone (5 μM) are shown by representative FACS plots. **(B)** Annexin V^+ve^ cells in different CD8^+^ T cell subsets are shown by bar diagrams. Mean and ± SD are shown. ***p ≤ 0.001 (Bonferroni test-Two-way ANOVA).

We showed an enhanced apoptosis of H-2K^b^-gB_495−505_-tetramer^−ve^CD44^hi^CD8^+^ T cells upon dexamethasone exposure (Figures 4C–F). This population constitutes an effector memory population as well. Therefore, we compared the expression levels of nr3c1 in naïve, activated as well as HSV1-specific CD8^+^ T cells in the memory stage at 60 dpi. Antigen reactive H-2K^b^-gB_495−505_-tetramer^+ve^CD44^hi^CD8^+^ T cells at 60 dpi expressed higher levels of nr3c1 as compared to those in the acute phase of the response and the expression levels were not significantly different from those in naïve cells (Figure 6F). The expression pattern of nr3c1 could also suggest that not only naïve but also memory cells could similarly respond to an exposure to dexamethasone. This could lead to an attrition of immunological memory generated in response to a previous infection or immunization as a consequence to dexamethasone therapy. We therefore evaluated whether dexamethasone causes apoptosis of memory cells generated during HSV1 infection. Draining LN cells were collected from HSV1 infected mice (30 dpi) and treated with 1 μM conc. of dexamethasone. In some of the wells mifepristone pre-treated cells were added. After 8 h of incubation, H-2K^b^-gB_495−505_-tetramer^+ve^ and H-2K^b^-gB_495−505_-tetramer^−ve^CD8^+^ T cells were analyzed for the frequencies of annexin V^+ve^ cells (Figures 7A,B). Virus specific memory CD8^+^ T cells (tetramer^+ve^CD8^+^ T cells) exhibited enhanced apoptosis (10.5 ± 1.2% in sham vs. 35.1 ± 2.5% annexin V^+^ in dexa group; 3 fold increase) after 8 h (Figure 7A upper panel, Figure 7B). Tetramer^−ve^CD8^+^ T cells also displayed greater apoptosis in similar experiments (40.3 ± 2.7% in sham vs. 73.4 ± 3.2% annexin V^+ve^ cells in dexamethasone group; >1.5 fold increase) (Figure 7A lower panel, Figure 7B).

The results therefore show that CD8^+^ T cells tightly regulate their nr3c1 expression during HSV1 infection. While the activated cells downregulate nr3c1 as compared to naïve cells, the memory cells regain the expression. The expression pattern of nr3c1 is directly correlated to their susceptibility to dexamethasone induced apoptosis. Naïve and memory cells are more prone to their elimination by even a transient dexamethasone therapy.

## Discussion

Immunosuppressive glucocorticoids are commonly used to control immunoinflammatory reactions caused by infections, autoimmune diseases and some cancers (6). The long lasting bioactive synthetic analogs of glucocorticoids act through cytosolic nr3c1 receptors expressed by most immune cells. The ligated receptor is translocated to nucleus to alter gene expression pattern in the responding cells to execute functions (8–12). We investigated the influence of a transient treatment of dexamethasone on differentiating virus-specific CD8^+^ T cells during herpesvirus infections. The virus-expanded effector CD8^+^ T cells resisted dexamethasone induced apoptosis. A greater propensity of naïve and memory CD8^+^ T cells to undergo dexamethasone-induced apoptosis as compared to virus-specific activated cells directly correlated with their higher nr3c1 expression levels. More of the persisting virus-specific CD8^+^ T cells produced effector molecules and preferentially homed to inflammatory tissue sites owing to their dexamethasone-induced upregulation of CD103 and CXCR3. These results therefore alert us on the limited utility of corticosteroids as the choice of immunosuppressant during viral infections.

The animals receiving dexamethasone therapy only transiently mounted a diminished anti-viral CD8^+^ T cell response later on due to a reduction in the precursor frequencies of naïve cells. This scenario could have considerable implication for patients with hematopoietic deficiencies where the output of specific CD8^+^ T is limited. Therefore, the available pool of naïve CD8^+^ T cells could be greatly reduced as a consequence of corticosteroid therapy. Similarly the use of immunosuppressive corticosteroids could further enhance the susceptibility of organ transplant patients or those with autoimmune conditions to multiple infections to which these patients might be exposed subsequently. Patients in these situations are usually prescribed with corticosteroids (2, 37). The observations could also imply that the prescription of corticosteroids in aged individuals might need to be carefully evaluated as their hematopoiesis and thymopoiesis is greatly compromised. The corticosteroid by depleting the precursor naïve cells and preexisting memory pool could make these individual exceedingly prone to multiple infections. That dexamethasone compromises the efficacy of vaccines was supported by a recent study. The patients given neoantigens based vaccination without dexamethasone treatment induce an infiltration of polyfunctional anti-tumor CD8^+^ T cells to the glioblastoma tumors, whereas the infiltration of such cells was greatly reduced in patients receiving dexamethasone during the vaccination (38). Conceivably, dexamethasone might have reduced the precursor frequencies of naïve CD8^+^ T cells that differentiate into effector and memory cells upon antigenic stimulation. Our findings that dexamethasone causes the depletion of a pre-existing virus-specific memory CD8^+^ T cells could also be of major interest to vaccinologists and clinicians. Thus, vaccinated individual maintaining a normal memory pool could potentially deplete their memory pool as a result of corticosteroid therapy. Memory cells reside in the host for longer duration to effect a quick protection against re-exposure with the same pathogen (39–41). If such a pool is reduced, the host could become greatly susceptible to infections.

We demonstrate a pronounced activity of viral reactive CD8^+^ T cells during an ongoing infection in animals receiving a transient dexamethasone treatment. This was evident both in terms of their enhanced ability to produce secretory effector molecules such as IFN-γ and CD107a, in addition to their up regulation of molecules such as CXCR3 and CD103 that facilitate cellular migration to inflammatory sites (Figures 1, 4, 6) (29). While both of these properties are considered favorable for CD8^+^ T cells in providing a better anti-viral defense but the intended effects of corticosteroid therapy would be limited. Thus, the anti-inflammatory regimens should dampen the activation status or help sequester effector cells in immune inductive sites in the lymphoid organs. Not only the chemokine receptor, CXCR3 triggers CD8^+^ T cells to migrate to inflamed tissue site but its expression along with that of KLRG1 is associated with short-lived effector cells (29–31). The expression of both CXCR3 and PD1 by activated cells would support such a notion as both molecules are associated with short-lived effector cells (31). A phenotype that is likely to give rise to tissue resident memory precursor cells was also evident among virus-specific CD8^+^ T cells in dexamethasone treated mice (Figures 2G–I). For many infections this population serves as the initial responder and eventually help resolve the infection quickly (13, 24, 42). How synthetic or endogenous glucocorticoids influence the functional memory pool is being investigated in our laboratory. Preliminary studies suggest for a preferential generation of a tissue resident memory population in animals receiving transient dexamethasone therapy (data not shown). The molecular details are being actively investigated in our laboratory.

It is well-known now that subsequent to a virus infection, the host becomes more susceptible to secondary infections and the potential reduction of granulocytes could account for such a susceptibility phenotype (43). Whether or not the elimination of naïve cells by the activity of endogenous glucocorticoids could contribute to the enhanced susceptibility of host to subsequent infections remains less explored and could indeed serve as one of the key factors. Glucocorticoid levels in the microenvironment could also potentially help dictate a specific differentiation program in CD8^+^ T cells and possibly other cell types as well. The relative expression levels of transcription factors Tbet and Eomes, regulates differentiating effector and memory cells populations during some infections (32, 44, 45). Thus, CD8^+^ T cells isolated from dexamethasone treated animals did not show alteration in Eomes mRNA levels but that of Tbet was greatly upregulated. However, a thorough investigation into the molecular pathways induced in dexamethasone exposed cells are likely to enrich our understanding. Many of these issues are currently under investigation. A very recent study implicated the role of endogenous glucocorticoids in NK cells activation during some herpesvirus infections (46, 47). Not only during pathological but also during physiological situations the levels of corticosteroids are modulated and whether this could serve as one potential variable in T cells speciation program to subsequent infection could also be a topical issue for investigation.

**Figure 8:**
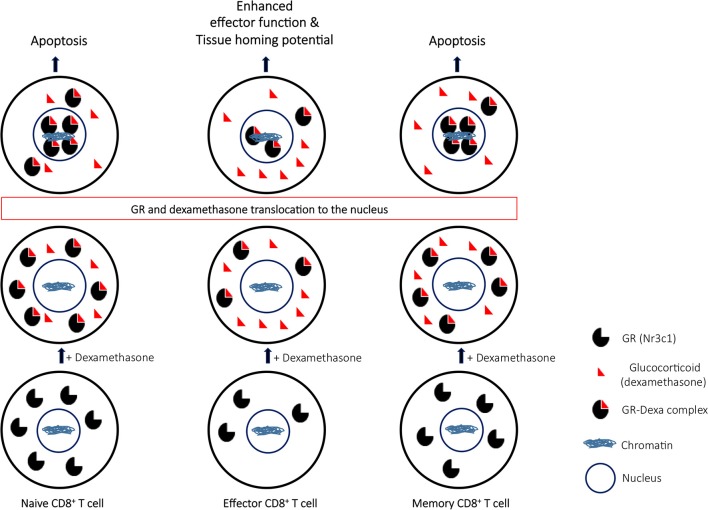
A proposed model to elucidate the role of glucocorticoids during differentiation of CD8^+^ T cells. Naïve CD8^+^ T cells upon their activation downregulate the nr3c1 receptor. However as the effector cells further differentiate to become memory cells, nr3c1 receptor is up regulated. Naïve and memory cells owing to their enhanced expression for glucocorticoids undergo dexamethasone induced apoptosis while the effector cells only have limited pool of nr3c1 available for ligating to dexamethasone. The receptor ligation induces translocation of ligated nr3c1 to nucleus where the relative abundance of such complexes dictates the cell function and fate by transcriptional regulation.

Our study has several implications. First, dexamethasone augmented tissue homing and effector potential of CD8^+^ T cells, thereby limiting its utility as an acute anti-inflammatory agent. Second, dexamethasone could enhance susceptibility to subsequent infection due to depletion of naïve CD8^+^ T cells. Finally, dexamethasone could cause attrition of pre-existing immunological memory generated either as a result of natural infection or vaccination as summarized in Figure 8.

## Data Availability

The raw data supporting the conclusions of this manuscript will be made available by the authors, without undue reservation, to any qualified researcher.

## Ethics Statement

Institutional Animal Ethics Committee (IAEC) of IISER Mohali set up by the Committee for the Purpose of Control and Supervision of Experiments on Animals (CPCSEA), which is established under Chapter 4, Section 15(1) of the Prevention of Cruelty to Animals Act 1960. IAEC approved all the animal experimental protocols. The numbers of protocols are IISERM/SAFE/PRT/2016/09 and 011. All the procedures performed were strictly according to the approved protocols.

## Author Contributions

DK performed the experiments, designed the study, wrote the manuscript. SS designed the study, interpreted data, wrote and edited the manuscript.

### Conflict of Interest Statement

The authors declare that the research was conducted in the absence of any commercial or financial relationships that could be construed as a potential conflict of interest.

## Supplementary Material

Figure S1 Effect of glucocorticoid on immune cells. The leukocytes and myeloid cells were measured in blood circulation of sham and dexa treated mice. (Experimental plan discussed in Figure 1). The frequencies of CD45^+^ cells **(A,B)** and their numbers **(C)** are shown. The frequencies of CD11b^+^ myeloid cells **(A,D)** and their numbers **(E)** in PBMCs are shown at 6 dpi (ref Figure 1 legend). Each symbol shows an individual animal, where error bars indicate ± SD. ^**^*p* < 0.005; ^***^*p* < 0.001 and NS (*p* > 0.05)- not significant (Mann-Whitney *U* test- two tailed).

Figure S2 Effect of different doses of dexamethasone on virus specific CD8^+^ T cells. C57BL/6 mice (*n* = 15) were infected with HSV1. At 4–6 dpi the animals were treated with sham (3 mice), and graded dose of dexamethasone (10, 0.1, and 0.01 mg/kg−4 mice in each group) through intraperitoneal route. On 6 dpi the cell types in blood circulation were counted. Frequency **(A,B)** and count/ml **(C)** of CD8^+^ T cells are shown by bar graphs. The percentage **(D,E)** and count/ml **(F)** of H-2K^b^-SSIEFARL-tetramer^+ve^ CD8^+^ T cells are shown. **(G)** The frequencies of CXCR3^+^ H-2K^b^-SSIEFARL-tetramer^+ve^ CD8^+^ T cells are shown. Levels of HSV reactive cells at memory stage and recall infection were measured. Frequency and count **(H–J)** of CD8^+^ T cells in blood circulation at memory stage (30 dpi) are shown. At 4 dpi of the recall infecti (with HSV1-KOS 2 × 10^6^ pfu/foot pad) the frequency and count of H-2K^b^-SSIEFARL-tetramer^+ve^ CD8^+^ T cells **(K–M)**, and count of CD8^+^ T cells **(N)** are shown. Each symbol shows an individual animal, where error bars indicate ± SD. ^*^*p* < 0.05, ^***^*p* < 0.001 and NS (*p* > 0.05)- not significant (Mann-Whitney *U* test- two tailed).

Figure S3 Measuring chemokine receptors in CD8^+^ T cells HSV1 infected mice that received either the diluent or dexamethasone. At acute stage of HSV1 infection CXCR4 and CCR7 expression on H-2K^b^-SSIEFARL-tetramer^+ve^ and H-2K^b^-SSIEFARL-tetramer^−ve^ CD8^+^ T cells in blood circulation **(A–E)** and draining popliteal LN **(F–J)** are shown. A Representative FACS plots and overlaid histograms for CXCR4 and CCR7 in H-2K^b^-SSIEFARL-tetramer^+ve^ and H-2K^b^-SSIEFARL-tetramer^−ve^ CD8^+^ T cells are shown from peripheral blood of sham and dexamethasone treated animals. The frequencies **(B,D)** and MFI values **(C,E)** for CXCR4 and CCR7 expression on H-2K^b^-SSIEFARL-tetramer^+ve^ or H-2K^b^-SSIEFARL-tetramer^−ve^ CD8^+^ T cells in peripheral blood are shown. **(F)** Representative FACS plots and overlaid histograms for CXCR4 and CCR7 in H-2K^b^-SSIEFARL-tetramer^+ve^ and H-2K^b^-SSIEFARL-tetramer^−ve^ CD8^+^ T cells are shown from draining popliteal LNs of sham and dexamethasone treated animals. The frequencies **(G,I)** and MFI values **(H,J)** for CXCR4 and CCR7 expression on H-2K^b^-SSIEFARL-tetramer^+ve^ or H-2K^b^-SSIEFARL-tetramer^−ve^ CD8^+^ T cells in draining popliteal LNs are shown. Data is represented as mean ± SD. NS (*p* > 0.05)- not significant (Mann-Whitney *U* test- two tailed).

Figure S4 mRNA expression on naive and activated CD8^+^ T cells. FACS plots show the sorted populations and the post sort purity **(A)** for different subsets of cells used in Figure 6. **(B)** Post sort purity of naive and H-2K^b^-SSIEFARL-tetra^+ve^ cells sorted from sham and dexa treated group mice is shown. Impact of dexamethasone treatment on mRNA level in naive and H-2K^b^-SSIEFARL-tetra^+ve^ cells for nr3c1 **(C)**, Bcl2 **(D)**, and Eomes **(E)** isolated from HSV1 infected mice. The qPCR was performed in triplicates. NS (*p* > 0.05)- not significant (Unpaired Student's *t-*test two tailed).
