# Supplementary figures and images for: Divergent Effects of a Transient Corticosteroid Therapy on Virus-Specific Quiescent and Effector CD8+ T Cells
Source: Front Immunol. 2019 Jul 12;10:1521. doi: 10.3389/fimmu.2019.01521 (PMC6639716; doi:10.3389/fimmu.2019.01521)

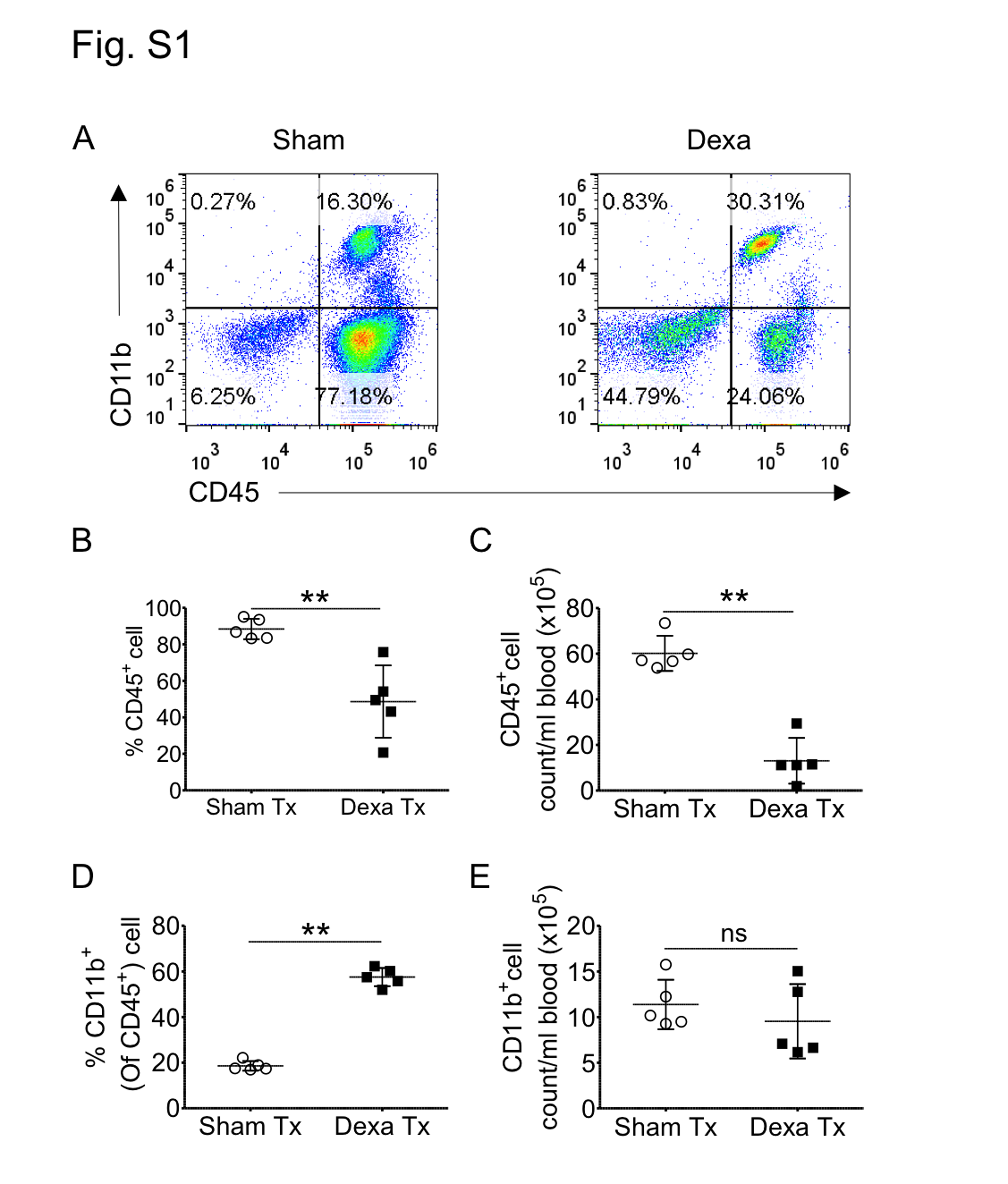

Supplement: Figure S1 — Effect of glucocorticoid on immune cells. The leukocytes and myeloid cells were measured in blood circulation of sham and dexa treated mice. (Experimental plan discussed in Figure 1). The frequencies of CD45+ cells (A,B) and their numbers (C) are shown. The frequencies of CD11b+ myeloid cells (A,D) and their numbers (E) in PBMCs are shown at 6 dpi (ref Figure 1 legend). Each symbol shows an individual animal, where error bars indicate ± SD. **p < 0.005; ***p < 0.001 and NS (p > 0.05)- not significant (Mann-Whitney U test- two tailed). [file Image_1.TIF]

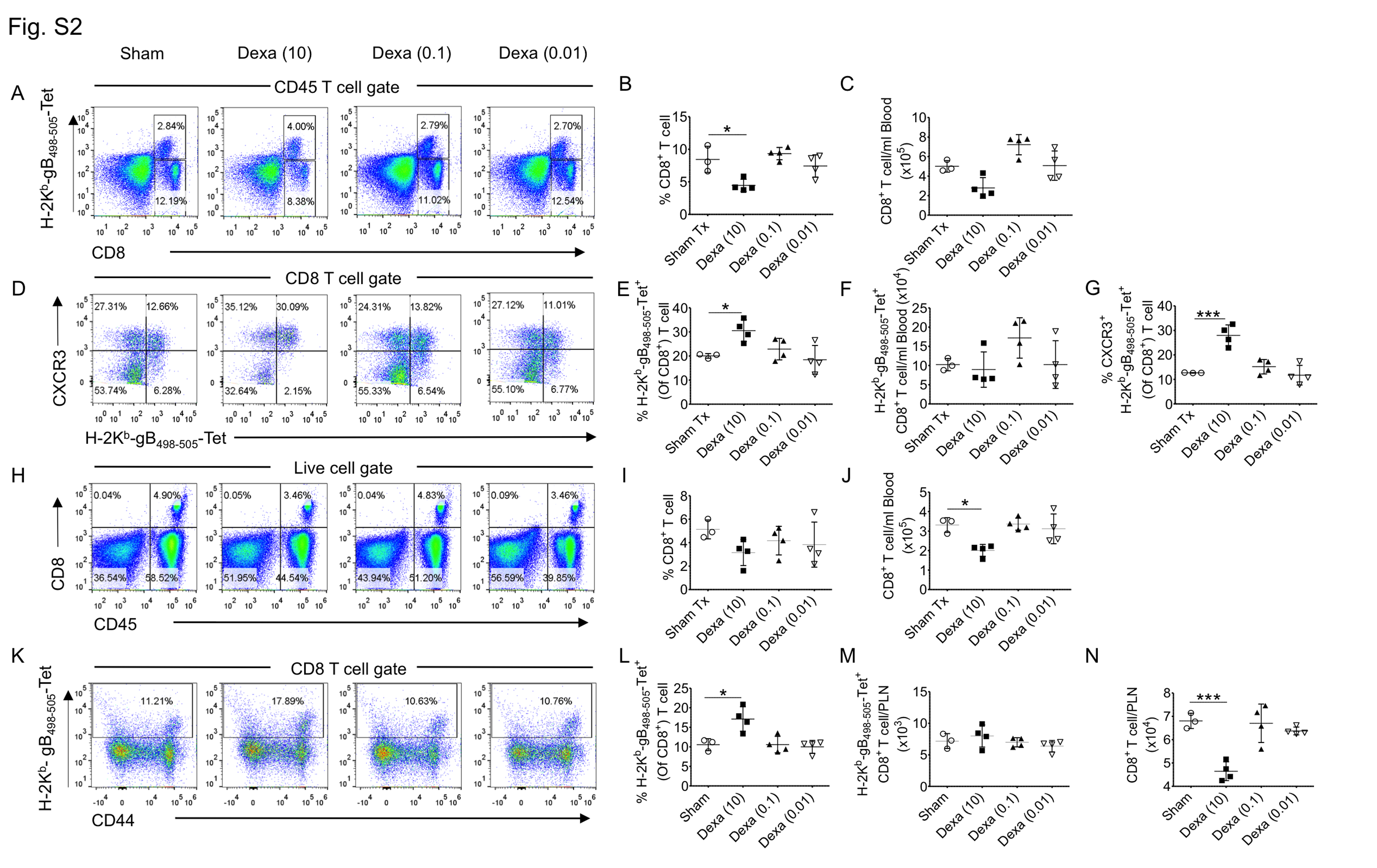

Supplement: Figure S2 — Effect of different doses of dexamethasone on virus specific CD8+ T cells. C57BL/6 mice (n = 15) were infected with HSV1. At 4–6 dpi the animals were treated with sham (3 mice), and graded dose of dexamethasone (10, 0.1, and 0.01 mg/kg−4 mice in each group) through intraperitoneal route. On 6 dpi the cell types in blood circulation were counted. Frequency (A,B) and count/ml (C) of CD8+ T cells are shown by bar graphs. The percentage (D,E) and count/ml (F) of H-2Kb-SSIEFARL-tetramer+ve CD8+ T cells are shown. (G) The frequencies of CXCR3+ H-2Kb-SSIEFARL-tetramer+ve CD8+ T cells are shown. Levels of HSV reactive cells at memory stage and recall infection were measured. Frequency and count (H–J) of CD8+ T cells in blood circulation at memory stage (30 dpi) are shown. At 4 dpi of the recall infecti (with HSV1-KOS 2 × 10^6^ pfu/foot pad) the frequency and count of H-2Kb-SSIEFARL-tetramer+ve CD8+ T cells (K–M), and count of CD8+ T cells (N) are shown. Each symbol shows an individual animal, where error bars indicate ± SD. *p < 0.05, ***p < 0.001 and NS (p > 0.05)- not significant (Mann-Whitney U test- two tailed). [file Image_2.TIF]

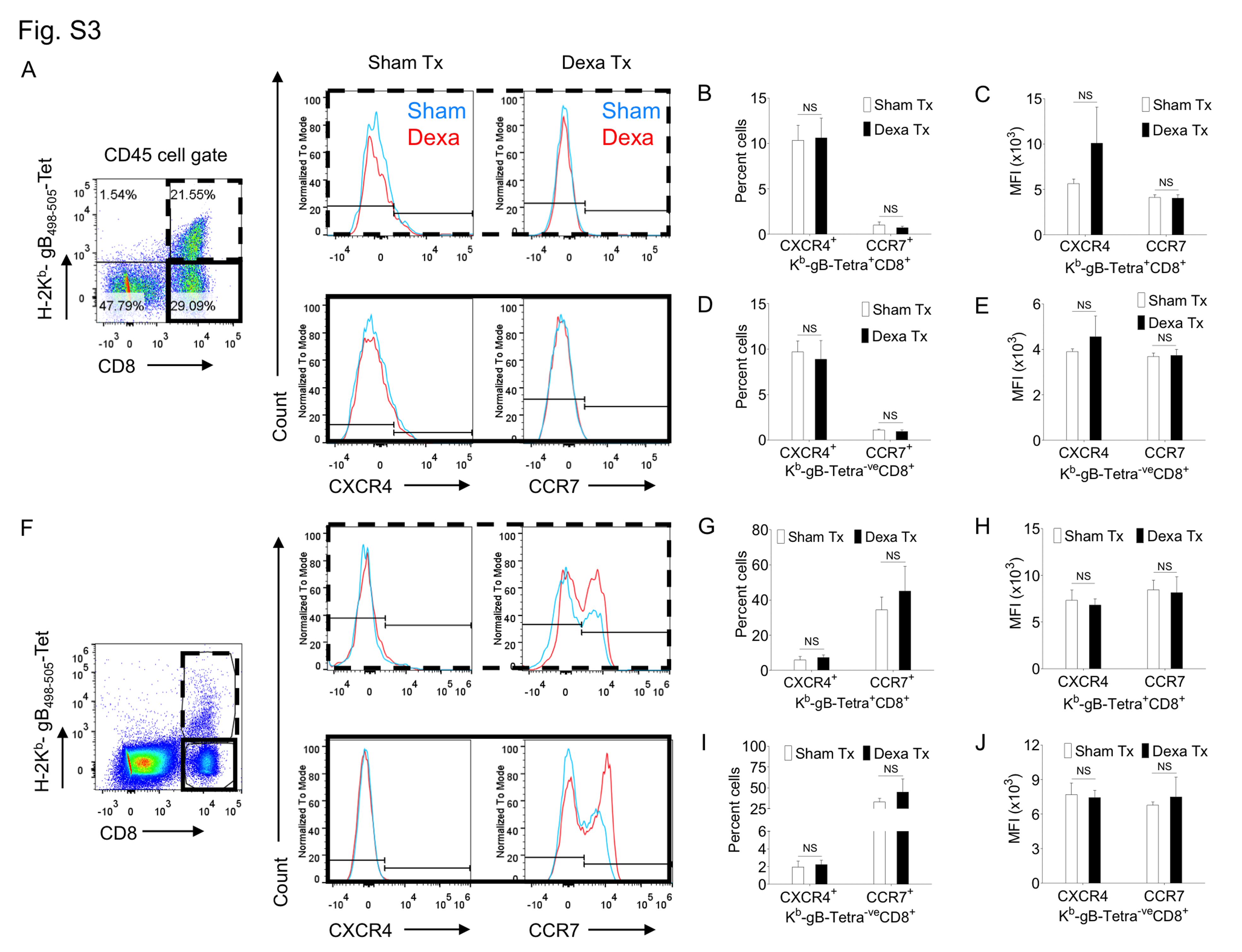

Supplement: Figure S3 — Measuring chemokine receptors in CD8+ T cells HSV1 infected mice that received either the diluent or dexamethasone. At acute stage of HSV1 infection CXCR4 and CCR7 expression on H-2Kb-SSIEFARL-tetramer+ve and H-2Kb-SSIEFARL-tetramer−ve CD8+ T cells in blood circulation (A–E) and draining popliteal LN (F–J) are shown. A Representative FACS plots and overlaid histograms for CXCR4 and CCR7 in H-2Kb-SSIEFARL-tetramer+ve and H-2Kb-SSIEFARL-tetramer−ve CD8+ T cells are shown from peripheral blood of sham and dexamethasone treated animals. The frequencies (B,D) and MFI values (C,E) for CXCR4 and CCR7 expression on H-2Kb-SSIEFARL-tetramer+ve or H-2Kb-SSIEFARL-tetramer−ve CD8+ T cells in peripheral blood are shown. (F) Representative FACS plots and overlaid histograms for CXCR4 and CCR7 in H-2Kb-SSIEFARL-tetramer+ve and H-2Kb-SSIEFARL-tetramer−ve CD8+ T cells are shown from draining popliteal LNs of sham and dexamethasone treated animals. The frequencies (G,I) and MFI values (H,J) for CXCR4 and CCR7 expression on H-2Kb-SSIEFARL-tetramer+ve or H-2Kb-SSIEFARL-tetramer−ve CD8+ T cells in draining popliteal LNs are shown. Data is represented as mean ± SD. NS (p > 0.05)- not significant (Mann-Whitney U test- two tailed). [file Image_3.TIF]

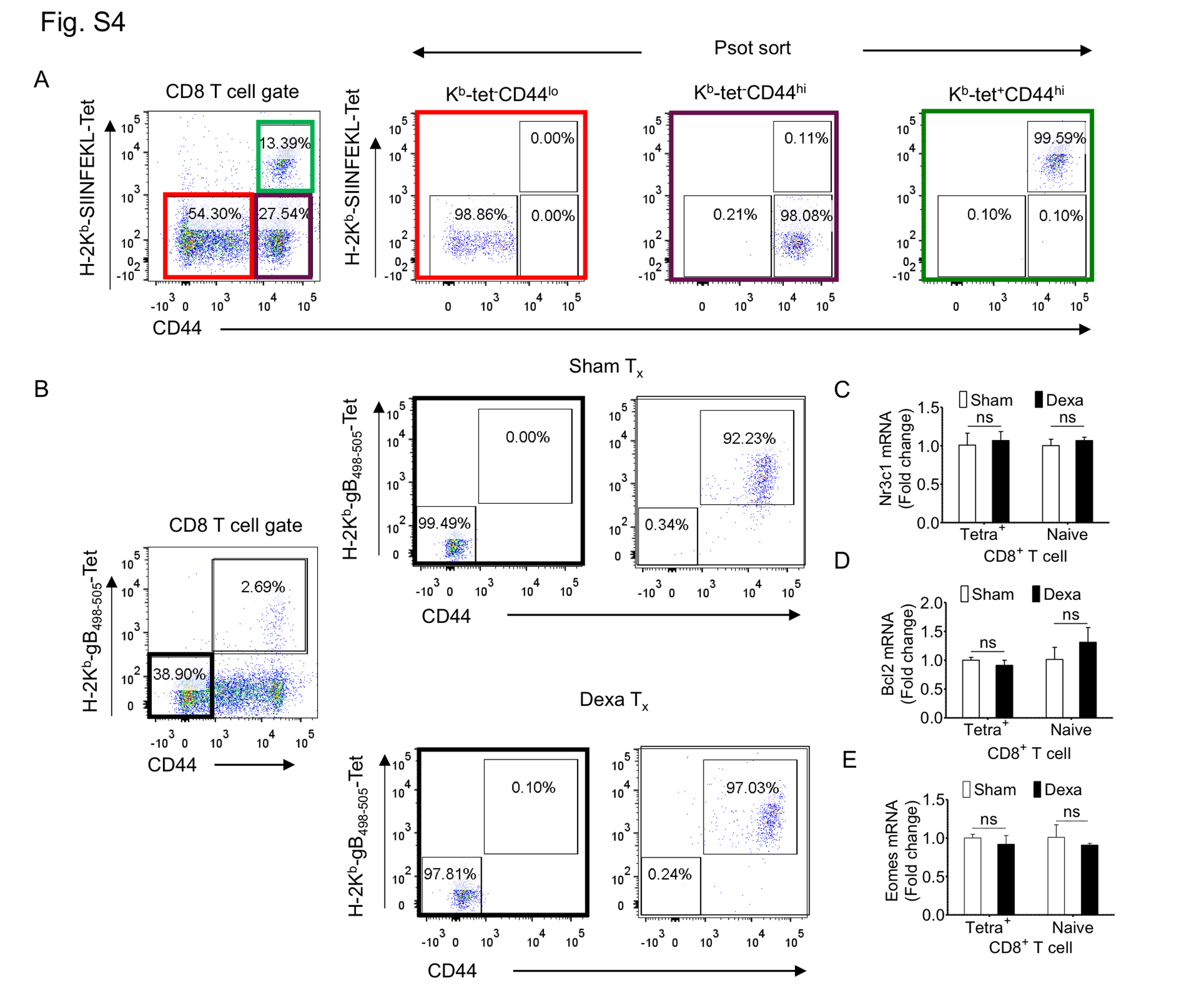

Supplement: Figure S4 — mRNA expression on naive and activated CD8+ T cells. FACS plots show the sorted populations and the post sort purity (A) for different subsets of cells used in Figure 6. (B) Post sort purity of naive and H-2Kb-SSIEFARL-tetra+ve cells sorted from sham and dexa treated group mice is shown. Impact of dexamethasone treatment on mRNA level in naive and H-2Kb-SSIEFARL-tetra+ve cells for nr3c1 (C), Bcl2 (D), and Eomes (E) isolated from HSV1 infected mice. The qPCR was performed in triplicates. NS (p > 0.05)- not significant (Unpaired Student's t-test two tailed). [file Image_4.TIF]
